# Web application for assisting non-dermatology physicians in learning and managing patients with common cutaneous adverse drug reactions: a multicenter randomized controlled trial

**DOI:** 10.1080/07853890.2024.2422573

**Published:** 2024-10-30

**Authors:** Kannattha Chaisriya, Weeratian Tawanwongsri, Dichitchai Mettarikanon, Nuchsara Ameentranon, Chime Eden, Mathat Inthongpan, Sasipaka Sindhusen

**Affiliations:** a Informatics Innovation Center of Excellence, School of Informatics, Walailak University, Nakhon Si Thammarat; b Division of Dermatology, Department of Internal Medicine, School of Medicine, Walailak University, Nakhon Si Thammarat, Thailand; c Division of Digital Content and Media, School of Informatics, Walailak University, Nakhon Si Thammarat, Thailand; d Su-Ngai Padi Hospital, Narathiwat, Thailand; e Jigme Dorji Wangchuck National Referral Hospital (JDWNRH), Bhutan; f Division of Digital Content and Media, Department of Digital Information Management, School of Informatics, Walailak University, Nakhon Si Thammarat, Thailand; g Bhumibol Adulyadej Hospital, Bangkok, Thailand

**Keywords:** Web application, drug eruptions, continuing medical education, knowledge, prognosis

## Abstract

**Background:**

Cutaneous adverse drug reactions (CADRs) remain a challenge for non-dermatologists. Medical-related applications to assist in learning about and managing patients with CADRs are scarce. We aimed to evaluate the efficacy of a web application for non-dermatologists in managing CADRs by comparing the knowledge scores of users and non-users.

**Materials and Methods:**

A multicenter randomized controlled trial was conducted between January 2023 and May 2023. Clinician participants were randomized (1:1) into the application and control groups using a simple randomization method. Knowledge scores between the groups were compared to evaluate the efficacy of the web application, and participants’ perspectives on the application were also collected.

**Results:**

A total of 44 clinician participants were included in the final analysis. The median age was 33.0 years (95% confidence interval (CI) 27.5–35.0) and predominantly female (56.8%). The score in the application group (median, 27.0; 95% CI, 25.0–28.0) was significantly higher than that in the control group (median, 14.0; 95% CI 13.0–17.0) (*p* < 0.001). There were no differences in scores between the sex groups (*p* = 0.695), between general practitioners (GPs) and non-GPs (*p* = 0.93), or among groups with different frequencies of evaluation of patients with CADRs (*p* = 0.266). In addition, the participants in the application group rated a high level of overall satisfaction.

**Conclusion:**

The web application for CADRs is an effective and convenient tool for assisting non-dermatologist physicians in learning and providing initial management with a high level of satisfaction. However, prospective long-term randomized controlled studies are required to confirm the efficacy of this tool.

## Introduction

Cutaneous adverse drug reactions (CADRs) are associated with health problems in both developing and developed countries, with incidences of 1–3 and 2–5%, respectively [1, 2]. Among adverse drug reactions, the skin is recognized as the most common organ involved (30–45%) [3]. The clinical spectrum of CADRs ranges from mild to severe. The World Health Organization (WHO) defines serious adverse drug reactions as any untoward medical occurrence that, at any dose, results in death, hospital admission, prolongation of existing hospital stay, persistent or significant disability/incapacity, or life-threatening consequences [4]. Adverse drug reactions contribute to hospitalization and mortality, with the rate of 0.1–0.15% and 5.64%, respectively [5, 6]. Potential risk factors include obesity, age > 60 years, immune dysregulation, and genetic predispositions [7–10]. The six common CADRs are maculopapular eruption, urticaria with or without angioedema, fixed drug eruption, acute generalized exanthematous pustulosis, drug reactions with eosinophilia and systemic symptoms, and Stevens-Johnson syndrome/toxic epidermal necrolysis [11–13]. The latter three conditions are categorized as severe CADRs [14]. The common culprit drug groups include antimicrobials (34.10%), anticonvulsants (32.88%), and anti-inflammatory drugs (21.51%) [11].

To manage patients with suspected CADRs, a comprehensive step-by-step approach is needed, including collecting a relevant clinical history, identifying all current medications with their temporal relationships with the occurrence of eruptions, defining the primary lesion (e.g. urticaria, erythematous papules, pustules), performing physical examination to specify the distribution of eruptions, mucosal involvement and associated signs (e.g. fever, lymphadenopathy, excoriations, internal organ involvement), constructing the differential diagnosis, as well as, analyzing literature and laboratory results [2, 15]. Specific training, particularly in dermatological examinations, is needed to gain experience and skills for accurate diagnosis, differential diagnosis, and proper management, resulting in a better prognosis [16, 17].

In Thailand, the health system faces numerous problems owing to the constraints of dermatologists, particularly in rural areas, and their high workloads [18]. Not every hospital has dermatologists available to evaluate and manage patients with suspected CADRs. Thus, non-dermatologists primarily deal with these conditions. It is intrinsically challenging for these patients to confidently formulate dermatological diagnoses and provide comprehensive management. To date, medical technology has become a promising tool for assisting in learning as well as the diagnostic and therapeutic processes of numerous conditions, including cutaneous malignancy, diabetes or decubitus ulcer, psoriasis, atopic dermatitis, and onychomycosis [19–21]. Nevertheless, there is a lack of application development for scaffolding clinical knowledge and skills, as well as aiding the evaluation and management of CADRs. We *ab initio* engaged with a broad research question (RQ): ‘What is the efficacy of the application for learning CADRs and assisting in managing patients with CADRs among non-dermatologist physicians?’ Following team discussions with patients and public involvement (PPI), the following RQs were developed:

1. **RQ1**: Does the web application enhance non-dermatologists’ learning about CADRs and assist them in managing patients with CADRs?
2. **RQ2**: What are the participants’ perspectives on this web application?

We developed the web application for postgraduate learning to assist in the management of patients with CADRs, aiming that the participants’ knowledge and skills were sharpened by guided questions and simplified knowledge of the six common types of CADRs in the web application. We aimed to scrutinize the efficacy of a web application for non-dermatologists in managing patients with CADRs by comparing the knowledge scores between participants who used the application and those who did not.

## Materials and methods

### Patient and public involvement

Our research team prioritizes patient and public involvement (PPI) in our studies. To develop our initial research concept and methodology, we concentrated on enhancing recruitment and retention, improving communication, and refining the informed consent process [22]. Similar to our previous study [23], we invited PPI participants through posters displayed in the dermatology waiting room at Walailak University Hospital. These participants were encouraged to join a closed social media group on Facebook. Through this group, they were consulted about research topics and priorities, focusing on their expected outcomes as service users of the health system. After discussing the proposed study design, suggestions were made regarding the functions of the web application. They agreed that the methods and interventions were appropriate because they acknowledged the burden of CADRs and the relative shortage of dermatologists, particularly in rural areas. They then helped distribute the results and highlighted the importance of proper CADR management within their informal groups.

### Web design and development

**Figure 1.**
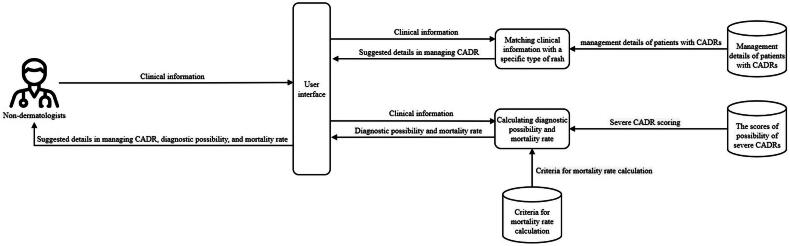
Conceptual framework of the web application for common cutaneous adverse drug reactions.

The conceptual framework was illustrated in Figure 1. The application matched patient information and a specific type of rash through the user interface screen. After processing, it chose the possible type of rash. A component of calculating diagnostic possibility and mortality rate processed after the user input the patient information. The application offered the suggested details consisting of the time interval between drug introduction and skin eruption, common culprit drugs, differential diagnosis, initial management, and prognosis—depending on the specific type of rash. It also allowed the user to copy and paste the suggested information into other applications or platforms. This, therefore, reduced the daily work burden of managing patients with CADRs.

We developed the web application to manage patients with CADRs under the supervision of an educational expert, a medical programmer, and three board-certified dermatologists. The web application was developed between July and December 2022. A programmer initially interviewed three board-certified dermatologists about the required essential components of the web application using a validated interview form (item-objective congruence index = 1.0) with a 5-point Likert scale ranging from ‘completely disagree’ to ‘completely agree’. After the system and data flow diagrams were created, the inputs and outputs were designed using a logical system. The web application, Vue.js (https://vuejs.org/), was selected as the intervention in this study because of its capacity of separating website elements, doing one function at a time while managing data in one file. Clinical knowledge obtained from the interview process was inserted, and the developed links and their functions were checked. The web application provided a simplified approach for CADRs with a step-by-step pattern. Its functions include displaying photos of six common CADRs with descriptions of skin lesions, the time interval between drug introduction and skin eruption, common culprit medications, possible causes, suggestions for initial management, and prognosis. For three severe CADRs, acute generalized exanthematous pustulosis, drug reaction with eosinophilia and systemic symptoms, and Stevens-Johnson syndrome/toxic epidermal necrolysis, a calculator for the possibility of these conditions and their severity were provided after filling out the patient information [24–26]. Three dermatologists reviewed the prototypes. A pilot study was conducted with a selected group of physicians from Walailak University. The web application was revised to a final version and implemented in Netlify (https://www.netlify.com/) in seamless coordination with Vue.js.

### Study design and population

This randomized controlled study was approved by the Walailak Ethics Committee (WUEC-23-012-01). Written informed consent was obtained from all clinician participants after receiving a full explanation of the study. This study complied with the principles of the Declaration of Helsinki and the International Conference on Harmonization of Good Clinical Practice. The ethics committee took into account and complied with Thailand’s laws, including the Personal Data Protection Act (PDPA). This study complied with the principles of the Declaration of Helsinki and the International Conference on Harmonization of Good Clinical Practice. We recruited clinician participants from four medical centers including Walailak University Hospital, Bhumibol Adulyadej Hospital, Naradhiwas Rajanagarindra Hospital, and Thasala Hospital. Data were collected between January 2023 and May 2023. We recruited clinician participants by inviting them to participate voluntarily through recruitment announcements at the four medical centers. The inclusion criteria required clinicians to be aged 18 years or older and to be non-dermatologists. The exclusion criteria included unwillingness to participate and incomplete questionnaires. To ensure confidentiality, all data files and sensitive personal information were encrypted, password-protected, and saved on a secure computer that was only accessible to the study coordinators. Clinician participants accessed their own data by directly contacting the study coordinators. No information linking individuals to the data was revealed. Twelve months after the completion of the study, all data were deleted.

### Intervention and assessment

**Figure 2.**
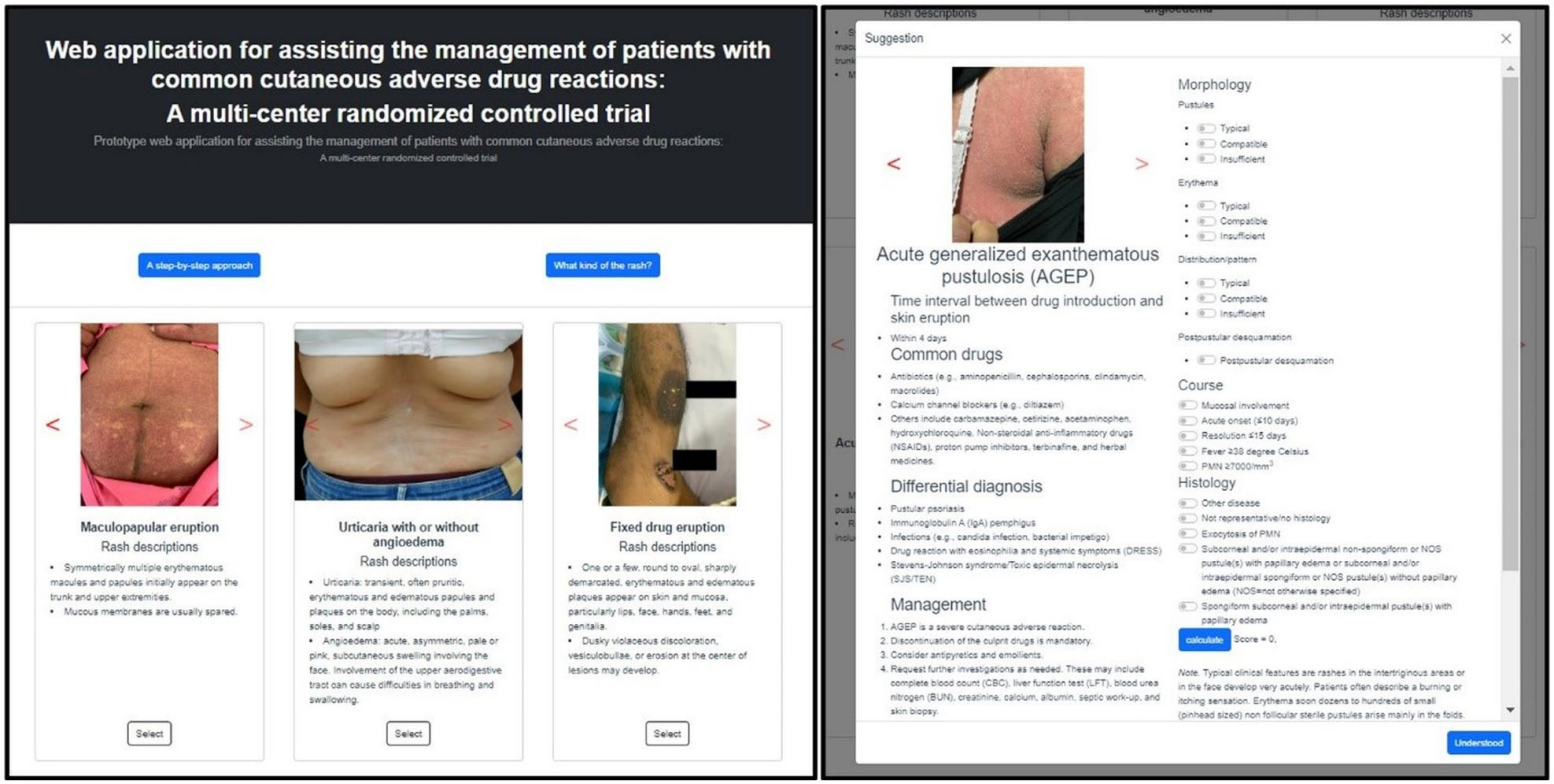
Selected screenshots of the web application used as the intervention in this randomized clinical trial.

The participants were randomized (1:1) with a simple randomization method using Microsoft Excel 2019 (Microsoft Corp., Seattle, WA, USA) into either the application group or the control group. The code sequence was kept in opaque sealed envelopes by non-medical staff who were not involved in the intervention. In the application group, participants were given a brief introduction to the web application, along with comprehensive instructions on its use, which took approximately five minutes. After this introduction, no further assistance was provided by the investigators. This application runs on a desktop computer with a 23.8-inch color screen (Hewlett-Packard Inc., Palo Alto, CA) on an internet website (https://research-rash.netlify.app). Selected screenshots are shown in Figure 2. All questionnaires and assessment measures were comprehensively reviewed and validated by three board-certified dermatologists and one educational expert using an item objective congruence (IOC) index. An IOC index score higher than 0.5 confirmed the content validity of the questionnaire [27]. A preliminary questionnaire was pilot tested with 20 volunteer non-dermatology physicians prior to the commencement of the study and revised for unclear wording. Internal consistency was assessed by calculating Cronbach’s alpha coefficient, and an alpha greater than or equal to 0.70 was considered acceptable [28].

Participants in both groups were asked to complete a questionnaire divided into two sections: Section 1 included participants’ characteristics, working experiences, and information sources together with the frequency they primarily searched; Section 2 consisted of six vignettes and 30 multiple-choice questions (MCQs) with five options related to the time interval between drug introduction and skin eruption, the most likely culprit medication, differential diagnosis, management, and prognosis. The maximum test score was 30. The participants were allowed 15 min to complete the MCQ section. The MCQ test was analyzed for difficulty index and power of discrimination. An average of the difficulty and discrimination indices of the 30 items were 0.67 (moderate difficulty) and 0.4 (very good discrimination), respectively. The participants in the application group were allowed to use the web application while taking the test, whereas those in the control group were instructed not to use any resources while taking the test. In the application group, participants were allowed to interact with this simplified interface application. This application offered a structured clinical approach to addressing CADRs, which was accessible *via* the ‘A step-by-step approach’ button on the initial webpage. Following this, users were required to either select the rash photo that best matched or identify the rash most similar to the descriptive symptoms or signs provided for each MCQ test. After selecting a specific rash, the application provided comprehensive information about the time interval between the introduction of the drug and the onset of the skin eruption, commonly associated medications, potential causes, initial management recommendations, and prognosis. Additionally, for three severe types of CADRs, the application included a calculator to evaluate the likelihood and severity of these conditions, which became available after the submission of relevant clinical information. The scores and correct answers with brief explanations were individually returned as feedback. At the end of the study, the participants in the application group were asked to complete a satisfaction survey on the web application. The scale ranged from 1 (do not agree) to 5 (completely agree). The knowledge scores of participants in both groups were compared to assess the efficacy of the web application.

### Statistical analysis

The sample size with an alpha of 0.05, power of 80%, and an estimated 15% loss to follow-up, calculated based on a previous study, was 22 in each group [29]. Continuous data are presented as means and standard deviations (SDs) or medians and ranges. Categorical data were presented as frequencies and percentages. The unpaired t-test or Mann–Whitney U-test was used to determine the statistical significance of the differences in knowledge scores. Associations between groups were determined using the Kruskal-Wallis, Mann-Whitney, or chi-square tests, as appropriate. Pearson’s correlation was used to determine the linear relationship between variables. Two-tailed tests were considered statistically significant at *p* < 0.05. Statistical analyses were performed using SPSS software version 18 (SPSS Inc., Chicago, IL, USA).

## Results

Of the 50 participants screened by a blinded investigator, 44 met the inclusion criteria and were invited to participate. There were no drop-outs during the trial. Participants, all of whom were physicians, were randomized into two groups. The median age was 33.0 years with a 95% confidence interval (CI) of 27.5 years to 35.0 years and a majority were female (56.8%). Most participants were non-general practitioners (GP) (59.1%) and worked at regional hospitals (43.2%) with a median work experience of 5.5 years (95% CI, 3.0–9.5). For GP physicians, the frequency of patients experiencing CADRs was as follows: never (5.6%), once or twice per week (72.2%), three to four times per week (16.7%), and seven times per week (5.5%). In contrast, for non-GP physicians, the frequency was: never (15.4%), once or twice per week (61.6%), three to four times per week (11.5%), five to six times per week (3.8%), and seven times per week (7.7%). Electronic resources (97.7%) were the primary sources of information used by physicians when evaluating patients. The participant demographics, comparing the application group and the control group, are summarized in Table 1.

**Table 1.** Characteristics of participants (*n* = 44).

Characteristics	Application group (*n* = 22)	Control group (*n* = 22)	p-value
Gender			0.761
Female, n (%)	13 (59.1)	12 (54.5)	
Male, n (%)	9 (40.9)	10 (45.5)	
Age, median (95% CI)	31.5 (27.0–35.0)	33.0 (30.0–36.0)	0.579
Working place			0.123
Private clinic/hospital	1 (4.6)	3 (13.6)	
Community (primary) hospital	3 (13.6)	3 (13.6)	
General (secondary) hospital	4 (18.2)	11 (50.0)	
Regional (tertiary) hospital	14 (63.6)	5 (22.8)	
Specialties			0.245
General practitioner	12 (54.6)	6 (27.3)	
Internal medicine and subspecialties	5 (22.7)	4 (18.2)	
Surgery and subspecialties	0 (0.0)	1 (4.5)	
Pediatrics and subspecialties	1 (4.5)	3 (13.6)	
Others*	4 (18.2)	8 (36.4)	
Years of working experience, median (95% CI)	3.0 (3.0–10.0)	8.0 (3.0–9.0)	0.427
Information sources they primarily consult when evaluating patients			0.999
Electronic resources, n (%)	22 (100)	21 (95.5)	
Paper-based resources, n (%)	2 (9.1)	2 (9.1)	
Human resources (specialists), n (%)	1 (4.5)	1 (4.5)	
Frequency of evaluating patients with CADRs			0.429
Never	1 (4.5)	4 (18.2)	
1–2 times per week	14 (63.7)	15 (68.2)	
3–4 times per week	4 (18.2)	2 (9.1)	
5–6 times per week	1 (4.5)	0 (0.0)	
≥ 7 times per week	2 (9.1)	1 (4.5)	

Note. CADRs, Cutaneous adverse drug reactions; CI, Confidence interval. *Others include radiology, emergency medicine, family medicine, orthopedics, ophthalmology, rehabilitation, and preventive medicine.

**Figure 3.**
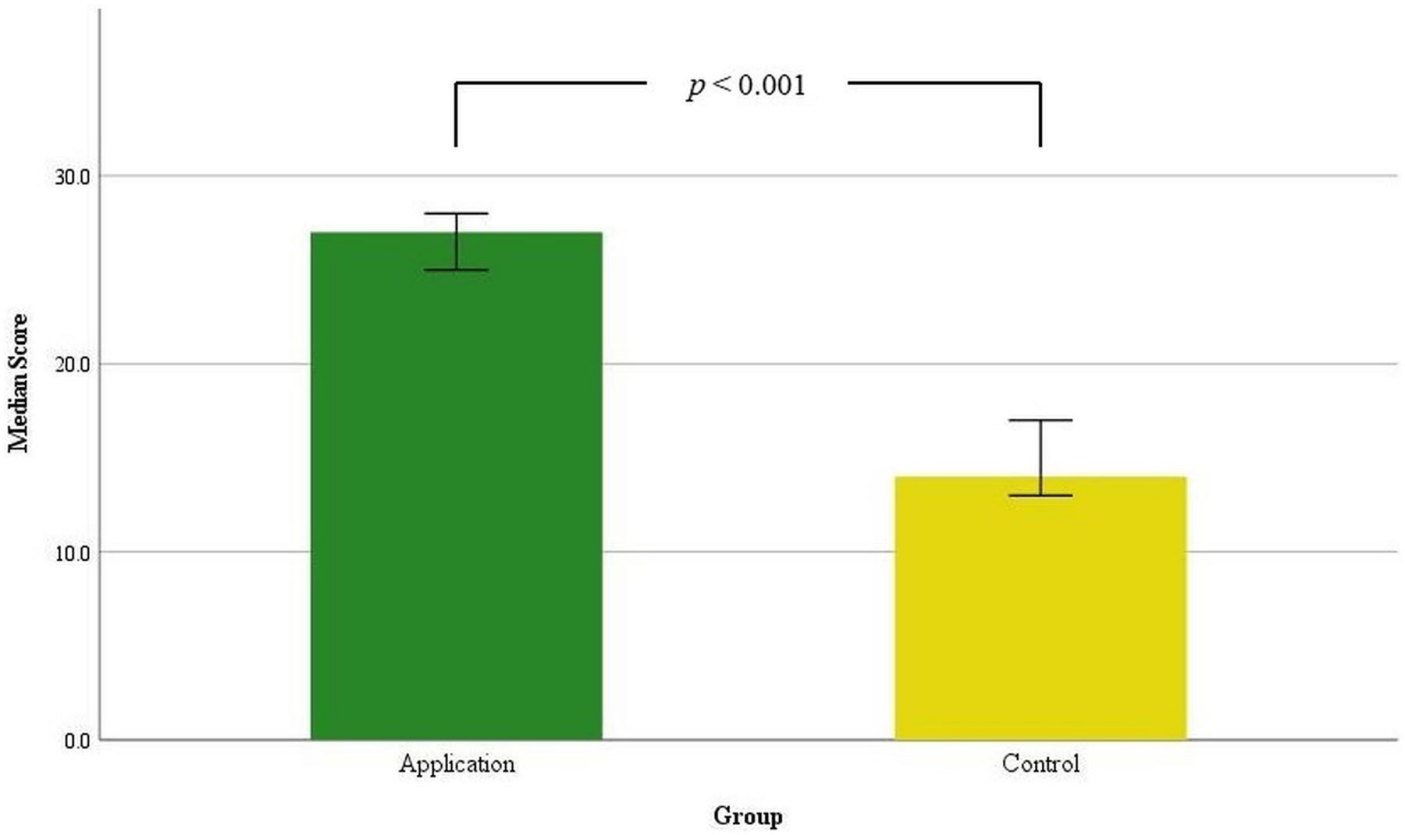
Efficacy of the web application for learning and managing cutaneous adverse drug reactions, represented by median scores in a comparison between the application group (*n* = 22) and the control group (*n* = 22), including 95% confidence intervals.

**Figure 4.**
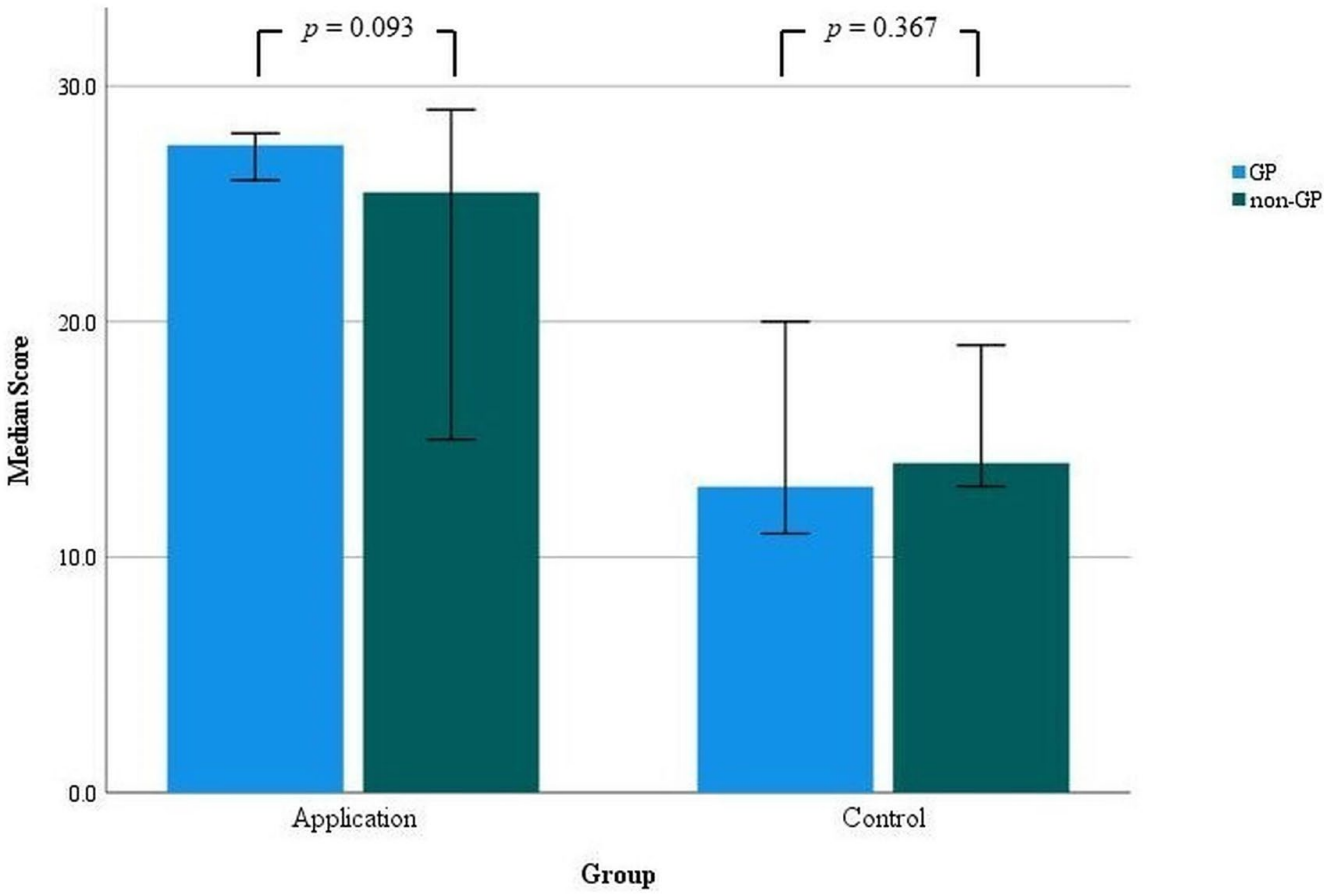
Comparative efficacy analysis in subgroups; general practitioner (GP) participants and non-GP participants across the application group (*n* = 22) and the control group (*n* = 22), including 95% confidence intervals.

The knowledge score in the application group was significantly higher (median, 27.0; 95% CI, 25.0–28.0) than in the control group (median, 14.0; 95% CI, 13.0–17.0), as shown in Figure 3 (*p* < 0.001). The median knowledge score in the GP group was 26.5 (95% CI, 16.0–28.0), which was significantly higher than the non-GP group’s median score of 16.5 (95% CI, 14.0–23.0), with a p-value of 0.035. There was no significant difference in scores between genders (*p* = 0.249) or among groups with different frequencies of evaluating patients with CADRs (*p* = 0.349). Additionally, there was no correlation between scores and participants’ age (*p* = 0.692) or years of work experience (*p* = 0.782). In the application group, there was no difference in scores between sex groups (*p* = 0.695), between the GP and non-GP groups (*p* = 0.093), or among groups with different frequencies of evaluating patients with CADRs (*p* = 0.266). There was no correlation between scores and age (*p* = 0.336) or between scores and years of work experience (*p* = 0.283). In the control group, there was no difference in scores between sex groups (*p* = 0.228), between the GP and non-GP groups (*p* = 0.367), or among groups with different frequencies of evaluating patients with CADRs (*p* = 0.273). We found a correlation between scores and participants’ age (r (22) = + 0.442, *p* = 0.040), and scores and years of working experience (r (22) = + 0.459, *p* = 0.032). Figure 4 illustrates the comparison of efficacy in the subgroup analysis between GP and non-GP groups within both the application and control groups. At the end of the study, the participants in the application group were asked to complete a satisfaction survey on the web application. The results indicated a high level of overall satisfaction. The detailed results are presented in Table 2.

**Table 2.** Participants’ perspectives on the web application (*n* = 22).

Evaluation Items	Mean	SD
**Design and format**		
It was convenient to find the CADR knowledge.	4.6	0.9
Fast page load times	4.6	0.7
The easy-to-read font type and size	4.5	0.8
The order of contents	4.6	0.9
The easy-to-use design	4.5	0.6
Modern and attractive design	4.3	0.7
The links functioned properly.	4.8	0.4
**Contents**		
The given content was clear, correct, reliable, and up-to-date.	4.7	0.5
The accordance of contents and clinical photos	4.8	0.4
Categorized contents allowed the users to easily search and understand the CADR knowledge.	4.8	0.4
Provided contents were essential and adequate.	4.6	0.5
**Usefulness and its application**		
The application was helpful for the application in patients with CADRs.	4.6	0.7
The application was a useful resource and reference as the users required.	4.7	0.5

Note. CADRs, Cutaneous adverse drug reactions; SD, Standard deviation. The scale ranged from 1 (do not agree) to 5 (completely agree).

## Discussion

Early recognition with proper management culminates in a better prognosis in patients with CADRs, particularly those with severe forms [30]. Due to the paucity of dermatologists or well-trained physicians in the care of patients with CADRs, particularly in rural areas in Thailand [18], coping with CADRs remains challenging. To the best of our knowledge, we first developed a web application for learning, together with assisting the management of patients with common CADRs, and evaluated its efficacy in a multicenter clinical randomized controlled trial among a wide range of non-dermatologist physicians, including general practitioners, specialists, and subspecialists. This web application provides clinical photographs of the typical clinical features of the six common CADRs, essential knowledge, and a comprehensive list of suggestions for management. It also helped users define the type of eruption based on their descriptions. We found significantly higher scores for knowledge of CADRs in the application group than in the control group. There were no differences in the scores between sex groups, between GPs and non-GPs, or among groups with different frequencies of evaluating patients with CADRs. Additionally, participants in the application group reported a high level of satisfaction with the web application.

Dermatology is a complex field of medicine. Dermatology residents receive training in clinical diagnosis and management through repetitive skin lesions and clinicopathological correlations to achieve their qualified competency. Without training or facilitated learning, non-dermatologists may find it difficult to manage patients presenting with eruptions [31, 32]. In addition, limited time is provided for learning dermatology in the undergraduate curriculum [33, 34]. Various medical applications have received increasing attention from physicians. Payne et al. [35] reported that medical students and junior physicians use various applications for medical procedures, diagnosis and management of diseases, calculation of clinical scores, and searching for drug references. Based on the significantly higher scores for knowledge of CADRs in the application group than in the control group, our web application with guided MCQs might be a promising learning tool to assist in learning. From the participants’ perspectives, the web application offered simplified and processed knowledge with related clear clinical photographs. It may even fit learners with a few years of work experience.

Continuing medical education is defined as an educational process that helps physicians fulfill their professional responsibilities in a more effective and efficient manner [36]. There are four components to maintaining standardized competencies: professional standing; self-assessment of knowledge, judgment, and skills; improvement in medical practice; and lifelong learning. Lifelong learning is required to empower physicians to obtain and maintain knowledge, attitudes, and skills [37]. Our findings support the benefits of self-directed learning through guided questioning, one of the most effective methods of lifelong learning. Dale’s Cone of Experience was modified into a learning pyramid [38]. This represents the level of experience with the instructional methods. The average learning retention rates for lectures, reading, audiovisual, demonstration, discussion, practice by doing, and teaching others were 5%, 10%, 20%, 30%, 50%, 75%, and 90%, respectively. Similarly, participants in the application group scored nearly twice as high as those in the control group *via* practice through simulation vignettes and reading the summarized knowledge provided on our web application.

Our web application offers essential and succinct clinical knowledge about CADRs that is suitable for various physicians and clinical settings, even in fast-paced situations. They are capable of gaining essential knowledge regardless of their specialty or work experience. Therefore, it ensures patient safety and drives the care standards for patients with CADRs. Likewise, Meyer et al. [29] revealed that a mobile application contributed to accurate diagnosis and appropriate laboratory test ordering in patients with coagulopathy. It also serves as a useful tool for learning and in real-world practice situations to improve patient outcomes. Our web application was designed to run on a website. Thus, it is easily approachable and flexible for self-directed learning and can be used in clinical settings. Like electronic books, they are preferred for searches, information retrieval, and convenience [39]. Therefore, attending physicians can make an initial critical decision for their patients whenever they face the problem of CADRs in real practice. Moreover, those who desired to improve their knowledge could comprehend the CADR topic through these guided questions in the test, core knowledge of the application, and correct answers with brief explanations as feedback. Our purpose in providing relevant, specific feedback is to promote reflection about their learning or performance, facilitate them in identifying areas to improve performance, and reinforce better performance with fewer repeated performance errors [40, 41].

Strengths of this study include an evaluation method on the efficacy of a web application based on the multicenter randomized controlled trial and no drop-outs. However, this study has several limitations. First, the varying extent of dermatology exposure among GP physicians may result in different baseline comprehension and a diverse range of preparedness in handling dermatological issues. Although our findings demonstrated no significant difference in scores among groups with different frequencies of evaluating patients with CADRs, this factor should be taken into account. Further studies should be conducted to assess the efficacy of the application among participants with varying levels of dermatology exposure. Second, our web application, which consists of six common CADRs, was designed to assist in the initial management of patients with suspected CADRs. Although it covered the majority of the CADRs, various other types of eruptions with dynamic and complex natures remained. Close follow-up rather than a one-point assessment is still crucial for CADRs. Thus, consultation or referral to well-trained physicians or dermatologists in the care of patients with CADRs is required to provide further definite suggestions. Third, we developed the web application and the MCQ test in English. This was the second language used by the participants. Their performance in web applications and tests may rely on their language competency. However, we tackled this problem by applying a simple language to web applications and tests. Fourth, the efficacy of the web application was evaluated using vignette-based questions. There is no evidence of its efficacy in the clinical management of patients, and these results may not reflect the application’s performance in real-world situations. Furthermore, we acknowledge that it is more convenient to use web applications on smartphones. Therefore, prospective long-term randomized controlled studies in both outpatient and inpatient units are needed to confirm the efficacy of web applications running on desktop computers and smartphones in reducing CADR-related morbidity and mortality. Fifth, our participants included relatively young clinicians, and randomization based on workplace and specialty was suboptimal. Before generalizing the study results, further research should confirm these findings through randomized trials involving clinicians of varying ages, diverse workplaces, and specialties. Sixth, the control group was restricted from accessing their usual information sources, simulating conditions where such resources were unavailable or scarce. Consequently, we observed significantly higher knowledge scores regarding CADRs in the application group compared to the control group. Moving forward, further studies are necessary to comprehensively evaluate the efficacy of our application compared to those using standard information sources, including electronic and paper-based resources, as well as other applications.

The web application for CADRs is an effective and convenient tool for assisting non-dermatological physicians in learning and providing initial management, with high levels of user satisfaction. However, further studies are needed to evaluate the actual efficacy of the application in guiding non-dermatologists in initial management. It is important to note that certain cutaneous eruptions are dynamic and complex. Therefore, consultation or referral to a dermatologist, along with close follow-up, is still necessary for comprehensive management.

## Data Availability

The data that support the findings of this study are available from the corresponding author, W.T., upon reasonable request.
